# Biomarker Prioritisation and Power Estimation Using Ensemble Gene Regulatory Network Inference

**DOI:** 10.3390/ijms21217886

**Published:** 2020-10-23

**Authors:** Furqan Aziz, Animesh Acharjee, John A. Williams, Dominic Russ, Laura Bravo-Merodio, Georgios V. Gkoutos

**Affiliations:** 1 Institute of Cancer and Genomic Sciences, Centre for Computational Biology, University of Birmingham, Birmingham B15 2TT, UK; f.aziz@bham.ac.uk (F.A.); j.a.williams@bham.ac.uk (J.A.W.); drr719@student.bham.ac.uk (D.R.); lxb732@student.bham.ac.uk (L.B.-M.); g.gkoutos@bham.ac.uk (G.V.G.); 2 Institute of Translational Medicine, University of Birmingham, Birmingham B15 2TT, UK; 3 NIHR Surgical Reconstruction and Microbiology Research Centre, University Hospital Birmingham, Birmingham B15 2WB, UK; 4 Medical Research Council Harwell Institute, Harwell Campus, Oxfordshire OX11 0RD, UK; 5 MRC Health Data Research UK (HDR UK), Midlands B15 2TT, UK; 6 NIHR Experimental Cancer Medicine Centre, Birmingham B15 2TT, UK; 7 NIHR Biomedical Research Centre, University Hospital Birmingham, Birmingham B15 2WB, UK

**Keywords:** gene regulatory network, causal modelling, omics integration, experimental design

## Abstract

Inferring the topology of a gene regulatory network (GRN) from gene expression data is a challenging but important undertaking for gaining a better understanding of gene regulation. Key challenges include working with noisy data and dealing with a higher number of genes than samples. Although a number of different methods have been proposed to infer the structure of a GRN, there are large discrepancies among the different inference algorithms they adopt, rendering their meaningful comparison challenging. In this study, we used two methods, namely the MIDER (Mutual Information Distance and Entropy Reduction) and the PLSNET (Partial least square based feature selection) methods, to infer the structure of a GRN directly from data and computationally validated our results. Both methods were applied to different gene expression datasets resulting from inflammatory bowel disease (IBD), pancreatic ductal adenocarcinoma (PDAC), and acute myeloid leukaemia (AML) studies. For each case, gene regulators were successfully identified. For example, for the case of the IBD dataset, the *UGT1A* family genes were identified as key regulators while upon analysing the PDAC dataset, the *SULF1* and *THBS2* genes were depicted. We further demonstrate that an ensemble-based approach, that combines the output of the MIDER and PLSNET algorithms, can infer the structure of a GRN from data with higher accuracy. We have also estimated the number of the samples required for potential future validation studies. Here, we presented our proposed analysis framework that caters not only to candidate regulator genes prediction for potential validation experiments but also an estimation of the number of samples required for these experiments.

## 1. Introduction

Network reverse engineering is the process of inferring the structure of a network from gene expression data through computational techniques. However, the problem of inferring the structure of a network is challenging for a number of reasons. The main challenge arises from the fact that while the number of genes in a given data set is high, typically the number of available samples is low. Additionally, since, in theory, all genes can potentially interact with each other, the number of interactions can be larger than both the number of genes and the number of available samples. It is also important to note that a gene regulatory network (GRN) is usually inferred directly from expression data that is, more often than not, noisy. For these reasons, it is highly unlikely that a single best method exists for every case [1,2]. Different methods highlight different interactions, and even the state-of-the-art methods generally achieve very low prediction accuracy [3].

Over the past two decades, several methods have been developed for GRN inferences. Most of these methods are based on unsupervised learning techniques and make different assumptions about the data used to generate GRNs. They usually exploit the statistical dependencies between genes so as to estimate the likelihood of existence of an interaction between two genes [2,4]. To that end, early methods typically used correlation coefficients to estimate the similarity between genes [5]. However, correlation coefficients suffer from the limitation that they fail to detect non-linear dependencies between the genes. Furthermore, since the correlation coefficient between two random variables X and Y is symmetric, i.e., corr$\left(X,Y\right)=$ corr$\left(Y,X\right)$, it cannot be employed to identify the direction of their interaction. In order to capture more complex dependencies between genes, analytical tools employing information theory, such as mutual information and entropy, have been widely used. For example, Butte et al. [6] have proposed a method that uses pair-wise mutual information (MI) to estimate the strength of a biological relationship between two genes. Since MI-based methods can detect many indirect links between genes, a number of refinements have been proposed to improve the prediction accuracy of the inference algorithm. For example, the ARACNE (Algorithm for the Reconstruction of Accurate Cellular Networks) [7] algorithm employs the Data Processing Inequality (DPI) method to filter out indirect interactions. CLR (Context Likelihood of Relatedness) [3] corrects the value of MI by comparing it with the empirical distribution of all mutual information scores in order to remove false correlations. MRNET [8] applies the Minimum Redundancy Maximum Relevance (MRMR) method [9,10] to rank direct interactions better than indirect interactions. Villaverde et al. [2] have proposed the MIDER (Mutual Information Distance and Entropy Reduction) approach, which uses mutual information and conditional entropies to infer the structure of a GRN. One of the advantages of MIDER is that it is general purpose and may be applied to any type of networks.

Recently, ensemble methods that formalise the GRN inference problem as a feature selection problem are becoming popular. For example, Huynh-Thu et al. [4] have proposed GENEI3 (GEne Network Inference with Ensemble of trees) that is based on variable selection with ensembles of regression trees. It decomposes the prediction problem into p different regression problems, where p is the number of genes. Next, the expression pattern of the target gene is predicted from the expression patterns of all the input genes using tree-based ensemble methods such as random forest or extra trees. GENEI3 achieved the highest performance in the annual DREAM (Dialogue for Reverse Engineering Assessments and Methods) In Silico Multifactorial challenge organised in 2009, namely DREAM4. Haury et al. [11] have introduced TRIGRESS (TRustful Inference of Gene REgulation using Stability Selection) that applies a different feature selection method, namely the least angle regression (LARS) method [12], combined with stability selection, to infer a GRN. Ruyssinck et al. [13] have proposed NIMEFI that generalises the GENIE3 regression decomposition strategy to other feature importance methods. However, NIMEFI requires more parameters to be adjusted than GENEI3. Guo et al. [14] have proposed partial least square based feature selection (PLSNET), an ensemble GRN inference method which is based on partial least squares (PLS). They have shown that PLSNET achieves higher accuracy on DREAM4 and DREAM5 (DREAM In Silico Multifactorial challenge organised in 2010) benchmarks when compared to other state-of-the-art methods. Furthermore, PLSNET is also efficient when compared to alternate methods [14].

In this paper, we aim to identify key regulators and infer the structure of a GRN from gene expression profile data. For this purpose, we have adopted two different methods, namely PLSNET [14], a feature-selection based method, and MIDER [2] (with small modifications), a method based on the mutual information between features. We demonstrate that, while the feature-selection based approach can successfully identify key regulators, the mutual information approach exhibits a better performance in inferring the structure of the network. We, therefore, propose a novel ensemble-based approach that combines the outputs of PLSNET and MIDER to infer the structure of a GRN that can be computationally validated. We demonstrate that the proposed ensemble-based approach can infer the structure of the GRN with higher accuracy, even for cases that both the PLSENT and MIDER fail. Furthermore, we have also estimated the number of the samples required to identify key regulator genes for potential future validation studies. The methods were applied to three different real-world datasets resulting from different disease studies, namely Inflammatory Bowel Disease (IBD) [15], pancreatic ductal adenocarcinoma (PDAC) [16], and acute myeloid leukaemia (AML) [17]. Additionally, we demonstrate the application of the ensemble-based approach to infer the structure of the network by applying it to the DREAM4 (Dialogue for Reverse Engineering Assessments and Methods) in silico network challenge. Finally, for each dataset, we analyse the structure of the inferred GRN, resulting from the application of MIDER, PLSNET, and our proposed ensemble method, and computationally validate our results using the loopy belief propagation algorithm (LBP) [18].

## 2. Results

### 2.1. Inflammatory Bowel Disease

**Figure 1:**
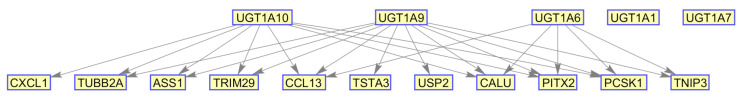
GRN generated from the application of partial least square based feature selection (PLSNET) on the inflammatory bowel disease (IBD) dataset.

Our first dataset pertains the gene expression profile resulting from an inflammatory bowel disease (IDB) study, that has 16 genes and 20 samples. In order to infer a GRN from this dataset, we first applied the PLSNET method. We used the same parameter values with the one proposed in the original study [14], namely m=4, T=1000, and $K=\sqrt{p}=4$, where p is the number of genes (See Section 4.2). Subsequently, we have chosen a threshold values that ensures that only 15% of the edges are selected. Figure 1 shows one of the possible GRNs produced by PLSNET.

The GRN produced by PLSNET shown in Figure 1 suggests that three genes, namely *UGT1A10*, *UGT1A9*, and *UGT1A6*, are the potential key regulators in this network. Note that, as explained in Section 4.2, PLSNET requires different parameter values to be adjusted. Among these, the threshold value, that determines how many edges are selected, is an important one. Choosing a different threshold value will result in the generation of a different network with different number of interactions. In addition, since PLSNET is stochastic, the PLSNET’s random initialisation of the regulatory genes may also result in the generation of different GRN with the same parameter values. Therefore, in every run of PLSNET, we expect a different GRN outcome. However, in our case we observed that, although the structure of the GRN in each iteration is different, the algorithm generally provides a clear separation between the target and the regulatory genes. This implies that the direction of the inferred interactions is usually drawn from a regulatory gene towards a target gene (or towards another regulatory gene).

Since each PLSNET run may result in different GRN outcomes, in order to increase the likelihood of correctly identifying different types of genes, we run PLSNET 100 times. For each run, we have identified the three different types of genes, i.e., regulatory genes (R), target genes (T), and intermediate genes (I). The regulatory gene is a gene with an in-degree of 0, while the target genes is a gene with an out-degree of 0. The in-degree of a gene in a network is the count of edges having this gene as a target gene and the out-degree is the count of the edges having this gene as a regulator gene. The intermediate genes are those genes that act both as a regulatory gene for some targets as well as target gene for some regulators. Furthermore, we have chosen different threshold values, such that, while maintaining a fixed set of parameters, only 2, 5, 10, 15 and 20% interactions are selected. Table 1 presents the frequencies of each gene acting as a regulatory gene, target gene, or intermediate gene for different threshold values. For small threshold values, our analysis has identified *UGT1A10* and *UGT1A9* as key regulatory genes. By decreasing the threshold value, we have further identified two more genes, namely *UGT1A6* and UGT1A7, as the potential key regulators. Note that the regulators of a UGT gene family, in the network produced by PLSNET, are always members of the UGT gene family, even when the threshold value is very small. One such network is shown in the Supplementary File (Figure S1), where a low threshold value is chosen such that 30% of the edges are selected.

**Figure 2:**
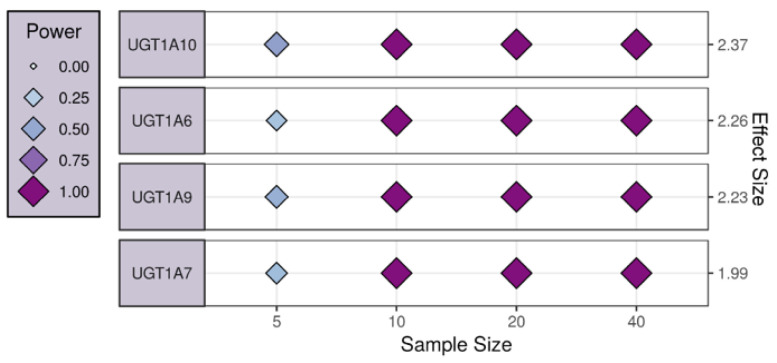
The four identified regulators for the IBD data are represented by the largest observed effect size. The effect size of each assessed variable is shown along the y axis and a series of sample sizes along the x axis.

We have also performed a power analysis using the IBD dataset. For this purpose, we have used the four genes, namely *UGT1A6*, *UGT1A10*, *UGT1A9*, and *UGT1A7*, that were identified by PLSNET as the potential key regulators, and estimated the number of samples required for the future validation experiments. Figure 2 represents the number of the estimated samples required for each regulator. For all the regulators the power achieved was by using 10 samples.

**Figure 3:**
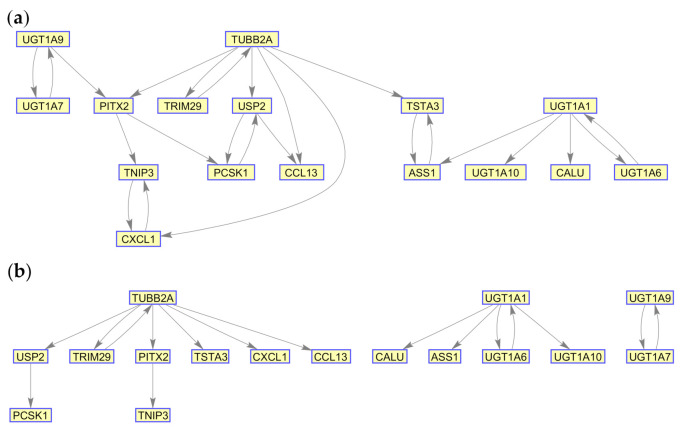
Gene regulatory network (GRNs) generated from the application of MIDER on the IBD Dataset. (**a**) GRN with all edges selected (no threshold). (**b**) GRN with selected edges (threshold corresponding to 65% edges).

We then applied the MIDER method using the same dataset. Similar to PLSNET, MIDER also accepts the input of different parameters. In our experiments, we have used the same values for all the parameters proposed by the authors in [2]. Figure 3a presents the GRN inferred by MIDER. Consistent to PLSNET, only members of the UGT gene family are identified as potential regulators of particular UGT genes. To analyse the structure of this network, we applied the loopy belief (LBP) [18] algorithm. The resulting low correlation coefficient (0.5533) implies that the marginal probabilities predicted by LPB on this GRN are not consistent with the observed states (marginal probabilities estimated from the data). In order to improve the accuracy of LBP, we gradually increased the threshold to discard edges with low scores. As a result, the accuracy of LPB increased until a certain threshold value was reached. For the threshold values corresponding to the selection of 80%, 70%, and 65% edges, LBP achieved correlation coefficient values of 0.7381, 0.9961, and ~1 respectively. The network corresponding to the selection of 65% edges, achieving a correlation coefficient of ~1, is presented in Figure 3b. Note that, since a very high correlation coefficient of ~1 was achieved, our proposed ensemble-based approach was not applied for this dataset. However, we observed that, the accuracy of the algorithm is further improved, if the edges from the network, whose target gene is a gene that has been identified as a potential regulator by PLSENT, are deleted. For example, for the network represented in Figure 3a, the correlation coefficient, upon introducing the output of PLSNET, was increased from 0.5533 to 0.7192. Similarly, for the Figure 3b network, the performance is increased, although the improvement is not significant (~5 × 10^−8^). Therefore, an ensemble-based approach can infer the structure of a network with higher accuracy than individual algorithms.

**Figure 4:**
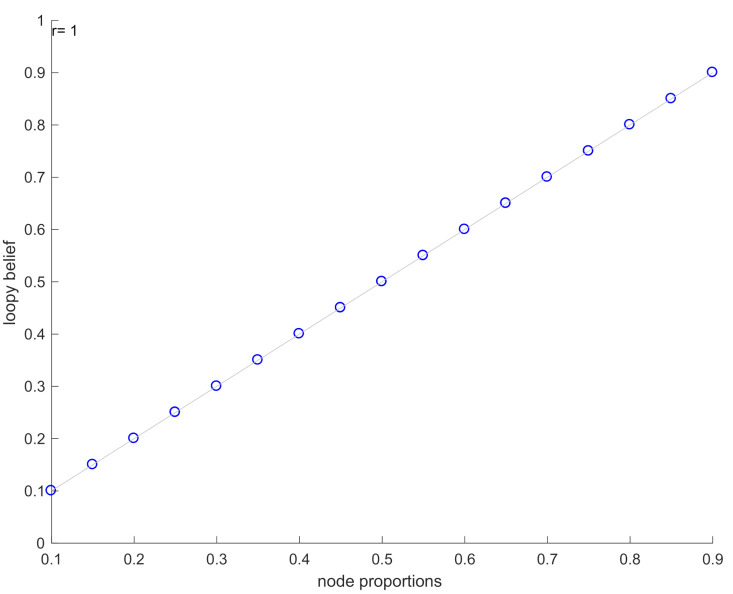
Pearson correlation plots for IBD dataset.

Table 2 presents the marginal probabilities predicted by the application of LBP algorithm to the GRN of Figure 3b and compare them with the probabilities estimated from the data. The loopy belief propagation algorithm, in this case, converges after 63 iterations. The results (Figure 4) reveal that there is a very high correlation (~1) between the model-predicted marginals and the observed experimental states.

### 2.2. Pancreatic Ductal Adenocarcinoma

**Figure 5:**
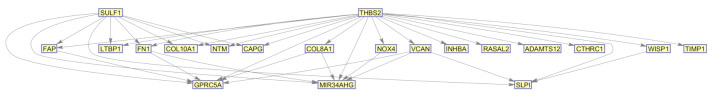
GRN generated from the application of PLSNET on pancreatic ductal adenocarcinoma (PDAC) dataset.

The pancreatic ductal adenocarcinoma (PDAC) microarray expression dataset (GSE15471) consists of 20 genes and 78 samples. In order to construct GRN from the data, we first applied PLSNET. We used the same parameters values as we have chosen in our previous experiment, i.e., we choose m=4, K=4 (we rounded off $\sqrt{20}$ to the nearest integer), and T=1000. Further, we have selected a threshold value such that only top 10% of the edges are selected. Figure 5 shows a GRN generated using PLSNET. Similar to the IBD dataset analysis, we run PLSNET 100 times for different threshold values and identified the three different genes types, i.e., regulatory genes, target genes, and intermediate genes. The results are presented in Table 3. The results identified two genes, namely *SULF1* and *THBS2*, as potential regulators. These genes were consistently selected as regulators when the threshold value is high and only 5% of the edges were selected. *THBS2* has a higher probability of being selected as a regulator gene and is predicted to have more interactions than *SULF1*. To estimate the number of samples required for further validation experiments, we have used the two key regulators, *SULF1* and *THBS2*, identified by PLSNET. For these two regulators, 5 samples estimated to be required for validation experiments. The estimated sample number required for each regulator is represented in the Supplementary Materials File (Figure S2).

**Figure 6:**
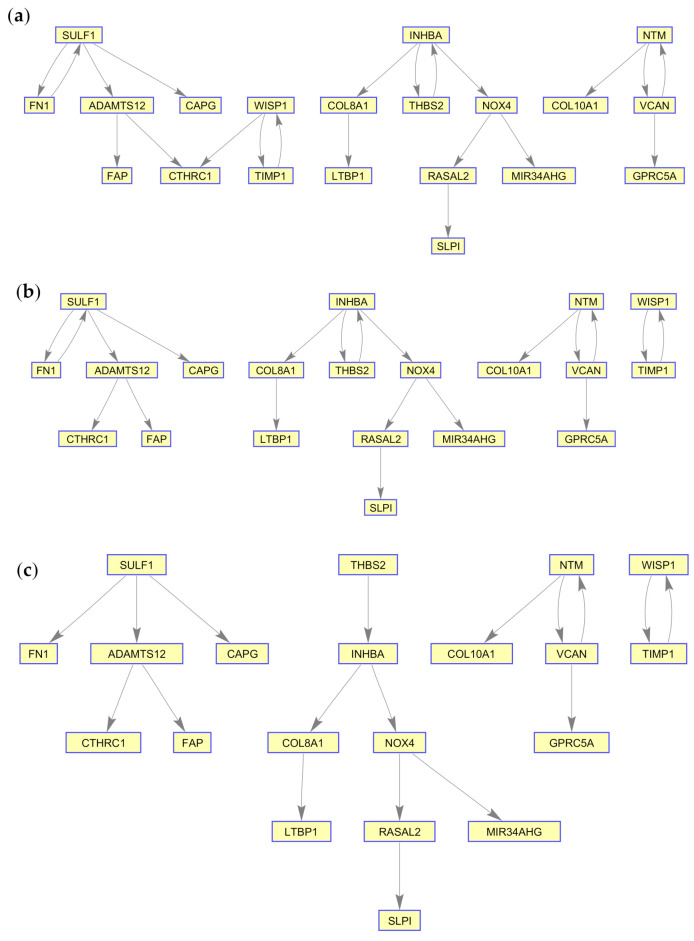
GRNs generated from the application of MIDER on the PDAC dataset. (**a**) GRN with all edges selected (no threshold); (**b**) GRN with selected edges (Using 95% threshold); (**c**) GRN with selected edges (Combining output of PLSNET).

**Figure 7:**
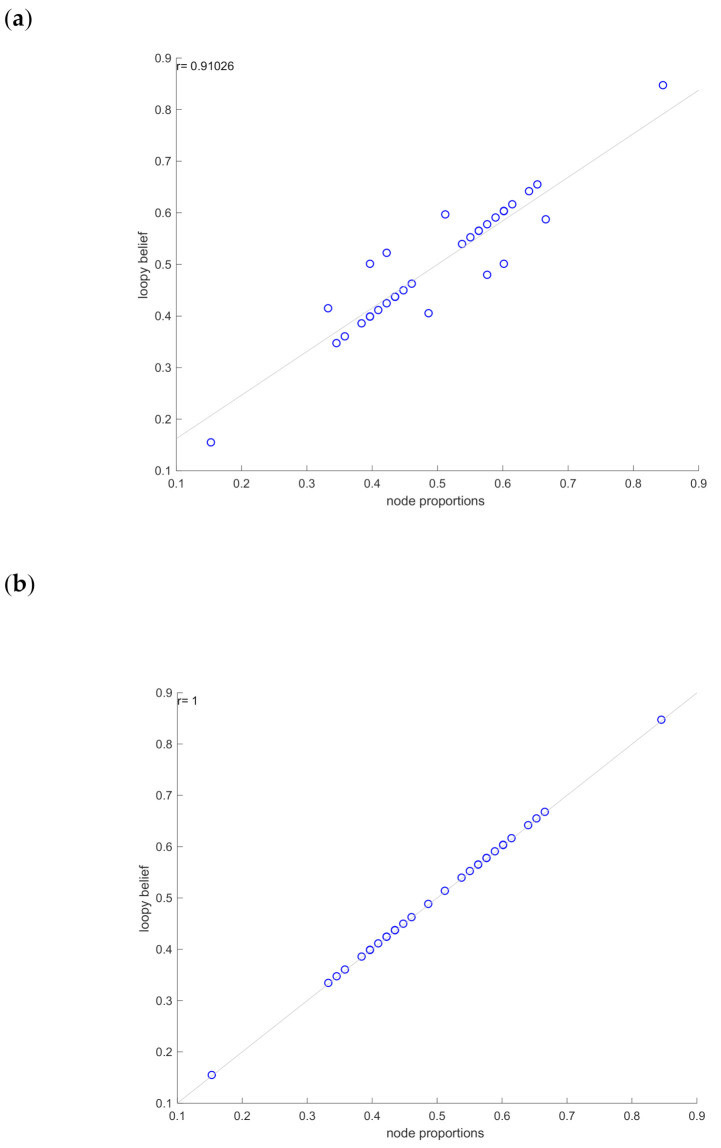
GRNs generated from the application of mutual information distance and entropy reduction (MIDER) on the PDAC dataset. (**a**) GRN with selected edges (95% threshold); (**b**) GRN with selected edges (Combining output of MIDER and PLSENT).

Although PLSNET catered the identification of the key regulators in the dataset, the network structure that was produced does not correspond well to the gene expression profile. This was validated by running the LBP algorithm on the PLSNET-generated GRNs with different threshold values, which resulted in a very low prediction accuracy. We subsequently assessed whether a MIDER-generated GRN over the same dataset would have a better network structure. The resulting GRN is shown in Figure 6a. In order to computationally validate our result, we applied the LBP algorithm on the network, which resulted in a low correlation value between the predicted marginals and the observed states. However, upon increasing the threshold value and further discarding 5% of the network edges, the results were significantly improved achieving a correlation coefficient of 0.9103. The resulting network is presented in Figure 6b and the values of the predicted marginals and the observed states for this case are plotted in Figure 7a. In order to determine whether the output of PLSNET and MIDER can be combined to produce a more accurate GRN, we identified and removed those network edges where the target gene is a gene that has been identified as a potential regulatory gene by PLSNET. Since, in our case, PLSNET identified *SULF1* and *THBS2* as potential regulators, we removed two edges from the network, namely the *ADAMTS12* to *SULF1* edge and the *INHBA* to *THBS2* one. Figure 6c presents that final network. The application of LBP over this network resulted in ~1 correlation coefficient. This increase in performance suggests that an ensemble-based approach is more effective in inferring the network structure than the individual approaches. The predicted marginals and the observed states are presented in Figure 7b. These estimated probability values can also be found in the Supplementary Materials (Table S1).

### 2.3. Acute Myeloid Leukaemia

The third dataset forms the largest gene expression dataset we considered and contains 60 genes and 542 samples from an acute myeloid leukaemia (AML) study (GSE15061). We first applied the PLSNET algorithm so as to identify the potential regulators within this dataset. The parameter values were the same ones used in the previous experiments. We set the threshold value that ensured that only the top 10% of edges were selected. The resulting GRN, suggesting that there is no single key regulator, is presented in the Supplementary File (Figure S3). Note that only 22 genes are presented in this figure. The remaining 38 genes have been omitted since they lack any predicted interaction participation. In order to gain a better understanding of the key regulators and target genes in this network, we applied the PLSNET algorithm 100 times with different threshold values. The frequencies of each gene, appearing as regulator, target, or intermediate, are given in Supplementary Materials (Table S2). The numbers reported in the table also suggest that there is no single gene that acts as a regulator within this dataset. There are few genes that sometime appear as regulators with very low probability, when the threshold value is very high. In this case we are discarding 98% of the edges. However, upon decreasing the threshold value, no regulatory genes were identified. Note that some genes in the Table S2 have zero cumulative frequencies for all the threshold values. Those genes lack any predicted interaction participation, even when the threshold value is very low.

**Figure 8:**
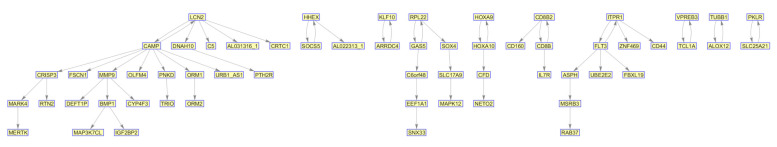
GRN generated from the application of MIDER on the acute myeloid leukaemia (AML) dataset.

In order to computationally validate the GRN generated from the application of PLSNET on the AML dataset, we applied the LBP algorithm to the resultant GRN. Similar to before, the LBP algorithm resulted to a very low correlation coefficient value between the predicted marginals and the observed states. To obtain a GRN that can be computationally validated, we applied the MIDER algorithm on this dataset. The MIDER based GRN contains 60 interactions and is presented in Figure 8. The LBP algorithm application over this GRN produced a very high correlation (with *r* ~ 1 and *p* ~ 0) between the predicted marginals and the observed states demonstrating that MIDER can accurately predict the observed gene states. The predicted marginal and the observed states are presented in the Supplementary File (Figure S4 and Table S3). Note that, since PLSNET has not identified any potential regulators within this dataset, the ensemble-based approach results in the same correlation coefficient (~1) for this case.

### 2.4. DREAM

For our last experiment, we have applied PLSNET, MIDER, and the ensemble-based model to benchmark networks generated for the DREAM4 in silico network challenge (http://wiki.c2b2.columbia.edu/dream/index.php/D4c2). We first selected a network with size 10 and containing105 samples. In order to identify key regulatory genes in this network, we applied the MIDER algorithm 100 times. The frequencies of each gene, appearing as regulatory, target or intermediate, is presented in the Supplementary File (Table S4) identifying ‘*G9*’ as the key regulator in this network. To infer the structure of the network, we next applied the MIDER algorithm to this data. In order to validate the GRN inferred from the application of MIDER, we have applied the LBP algorithm. The LBP algorithm achieved a correlation coefficient of 0.83913. In order to determine if the ensemble approach is more effective than the individual methods, we have deleted two network edges produced by the application of MIDER, one from the gene ‘*G7*’ to the gene ‘*G9*’ and the other from the gene ‘*G8*’ to the gene ‘*G9*’. These edges were deleted, since ‘*G9*’ was identified as a potential regulatory gene by PLSENT. The resultant GRN is presented in Figure S5 (Supplementary Materials). Upon validating this network, using LBP, a correlation coefficient of 0.97496 was achieved. This significant increase in performance clearly suggests that an ensemble-based model, combining the output of MIDER and PLSNET, has the potential to infer the structure of a GRN with a higher accuracy than the application of both the individual methods. The results are presented as Supplementary Materials (Table S5 and Figure S6). Similar to AML, PLSNET has not identified any regulatory gene in this network. When LBP algorithm was applied to the network inferred from the application of MIDER, a correlation coefficient of 0.8890 was achieved. Upon decreasing the threshold value and deleting further 40% of the edges from the network, the LBP algorithm has achieved a correlation coefficient of 0.9708.

### 2.5. External Biological Validation

**Figure 9:**
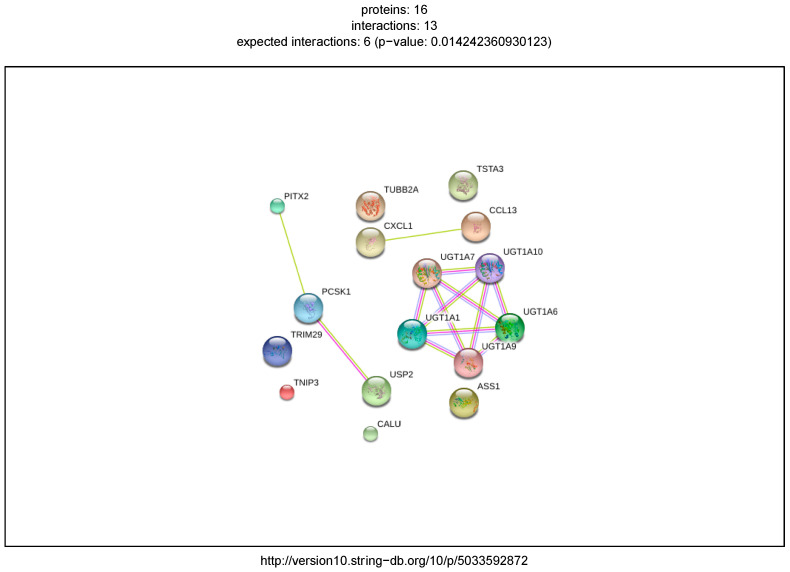
Predicted protein–protein interactions using the STRING database for the IBD GRN genes. Edges represent interactions between proteins, and multiple edges represent additional sources of evidence. Analysis was performed with String v. 10.

**Figure 10:**
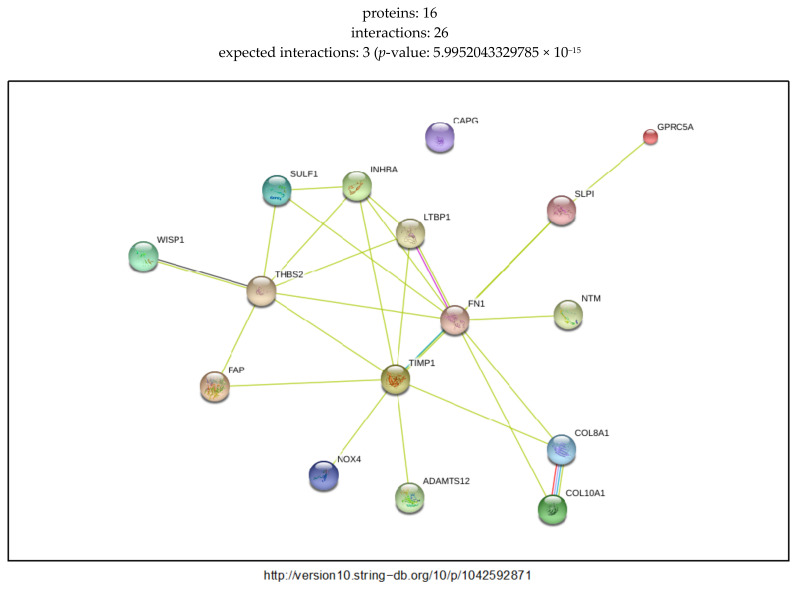
Predicted protein–protein interactions using the STRING database for the PDAC GRN genes. Edges represent interactions between proteins, and multiple edges represent additional sources of evidence. Analysis was performed with String v. 10.

Key regulatory transcripts and their putative targets from modelled IBD and PDAC GRNs showed varying degrees of biological plausibility when outside biomedical databases were consulted. The IBD regulator genes, all members of the UDP glucuronosyltransferase family, are highly enriched for flavonoid-substrate specific glucuronidation functions, see Figure S7. The enrichment analysis indicates involvement in bile secretion and ascorbate metabolism. The PDAC GRN is enriched for extra-cellular matrix degradation and integrin–protein binding processes, *TGF*-β and interleukin signaling, and cancer-specific pathways including glandular cell neoplasm formation, elevated carcinoma antigens, and senescence and autophagy in cancer (Figure S8). Protein–protein interactomes from the STRING database show higher than expected connectivity of the GRNs (*p* = 0.014 and *p* = 5.99 × 10^−15^, IBD and PDAC GRNs, Figure 9 and Figure 10).

**Figure 11:**
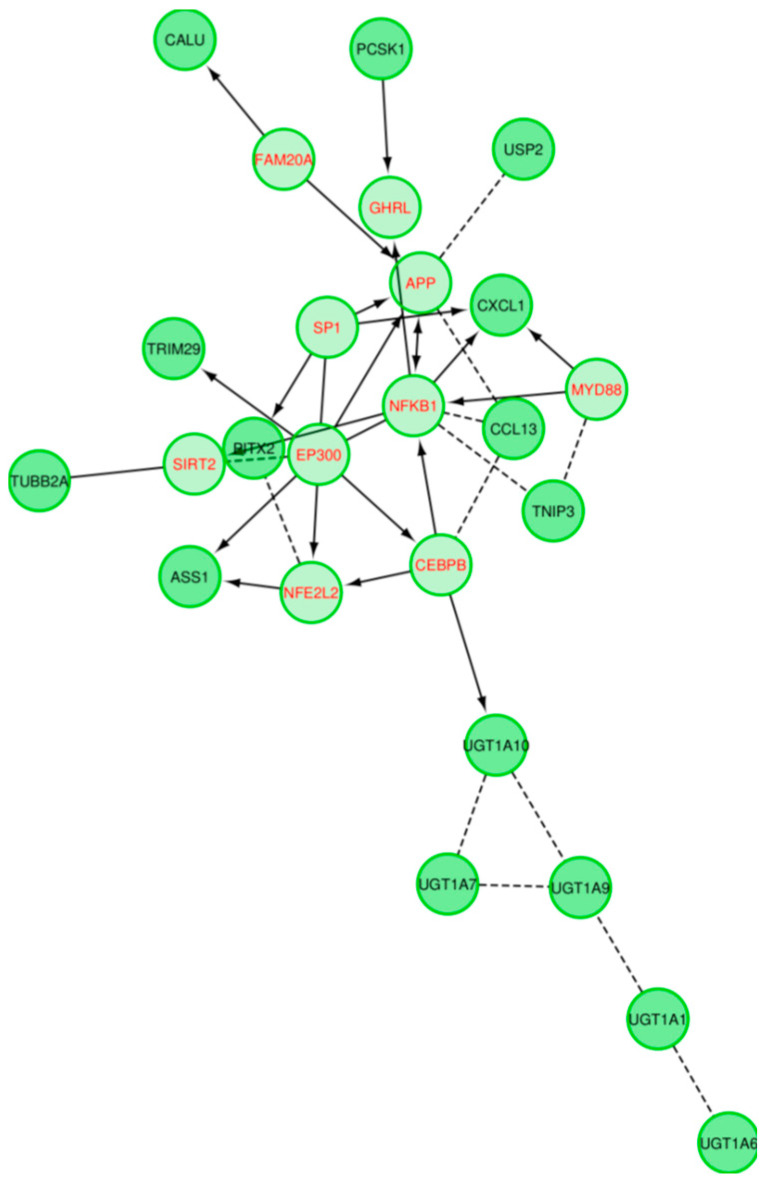
Reactome Functional Interaction visualization of the IBD dataset. Dashed edges are predicted associations. Directional edges indicate regulation, and T junction edges represent inhibition.

**Figure 12:**
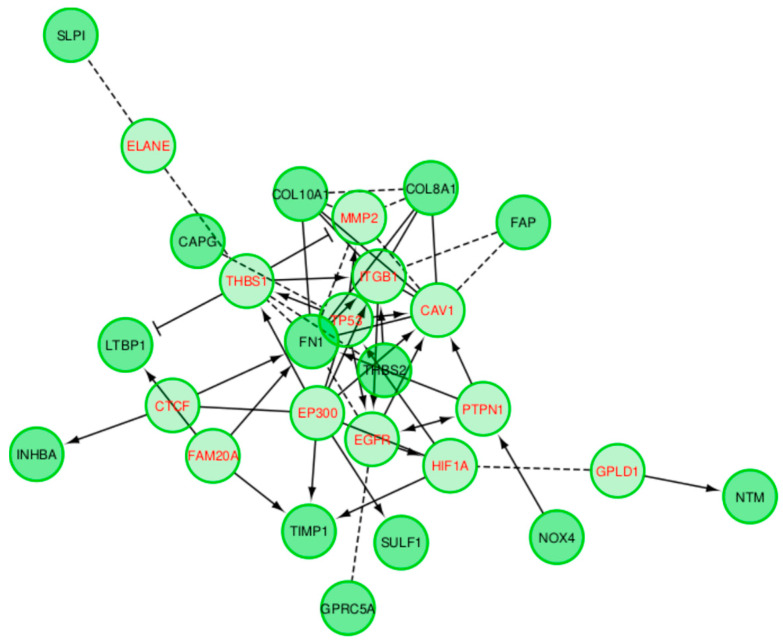
Reactome Functional Interaction visualization of the PDAC dataset. Dashed edges are predicted associations. Directional edges indicate regulation, and T junction edges represent inhibition.

Reactome-based functional interaction networks indicate sparse connectivity between core regulatory genes in the IBD dataset (Figure 11), while the PDAC GRN shows strong connectivity between one core regulator and neighboring genes (Figure 12).

## 3. Discussion

In this paper, we have explored the structure of a GRN inferred from the gene expression profiles of three different real-world datasets (IBD, PDAC, and AML) and one artificially generated dataset (DREAM4). To infer the GRN structure, we applied two different network inference algorithms, namely the PLSNET [14] algorithm and the MIDER [2] (with some modification) algorithm. This allowed us to identify key regulators, as well as analyse the structure of GRNs, amongst our datasets. Next, we have applied a novel ensemble approach, that combines the output of PLSNET and MIDER to infer the structure of a GRN with higher accuracy. We then computationally validated our results using the framework developed by Kotiang and Eslami [19] that applies the loopy belief propagation (LBP) [18] algorithm to predict gene states. Our analysis, across the three real-world, and the artificially generated datasets, suggests that the proposed ensemble method is more effective than both the MIDER and the PLSNET algorithms for inferring the structure of a GRN.

The results presented in Figure 1 and Table 1 demonstrate that PLSNET correctly identified all the *UGT* genes as potential regulatory genes within the IBD dataset. These results suggest that three genes, namely *UGT1A6*, *UGT1A10*, and *UGT1A9*, have a very high probability of making connections with other genes. In order to infer the structure of the GRN from the data, we next applied MIDER to the gene expression data. Although, by adjusting the threshold value, MIDER can infer the structure of the GRN with higher accuracy, we have observed that, for different threshold values, the accuracy can always be improved, if an ensemble approach that combines the output of MIDER and PLSENT is applied. In this case, the output of the MIDER was refined by deleting those edges from the network, where one of the three genes, *UGT1A6*, *UGT1A10*, and *UGT1A9*, appear as target genes and any gene other than these three genes are predicted as regulatory genes. Similar to the IBD dataset, the ensemble model is more effective than the individual methods when applied to the PDAC datasets. Our analysis shows that the application of the ensemble approach to the PDAC dataset achieves a correlation coefficient of ~1, which is significantly higher than the correlation coefficient achieved by LBP, when applied to the output of MIDER, as well as from the correlation coefficient resulting from the application of PLSNET on the same dataset. For the AML dataset, PLSNET has not identified any potential regulators and so the ensemble model results in a comparable performance in this case. Upon examining the DREAM4 dataset with 10 genes, we demonstrated that the performance of the LBP algorithm can be improved by using the ensemble model that combines the output of PLSNET and MIDER. In this case, we first produced a GRN by the application of MIDER and then deleted the false positive edges, identified by PLSNET, from the network. We demonstrate that LBP achieved a correlation coefficient of 0.97496 when applied to the output of the proposed ensemble method. For the same dataset, LBP achieved a correlation coefficient of 0.83913, when applied to the MIDER output. Finally, similar to the AML dataset, for the DREAM4 dataset with 100 genes, PLSNET has not identified any potential regulators in the network, and therefore the performance could not be further improved.

One of the weaknesses of the ensemble method adopted in this paper is that it requires us to adjust the values of the certain parameters before application. These parameters include the threshold value, required by MIDER to identify candidate edges from the network, and the threshold value, required by PLSENT to identify key regulatory genes in the network. To address these shortcomings, our future analysis will focus on automatically adjusting the threshold values used by the ensemble method to infer a computationally validated network from the data.

### Biological Relevance of IBD and DPAC Networks

The GRNs created for the two conditions, PDAC and IBD, were compared to existing biological networks created by non-gene expression sources. Of note, the IBD GRN was enriched for flavonoid gluconidation due to the presence of five *UTG1A* paralogs. All of the five core UGT genes, four of which are core regulatory genes in the GRN, interact with each other in the protein–protein interaction (PPI) network generated by STRING, with three sources of evidence driving a significant number of interactions, Figure 9. Notably, *UGT1A9*, *UGT1A1*, and *UGT1A6* are expressed in the liver and kidney cortex and basally expressed in the colon, while *UGT1A10* and *UGT1A1* are highly and specifically expressed in the transverse colon and the terminal ileum of the small intestine, indicating that these two proteins may indeed be core drivers of IBD-specific GRNs [20]. Multiple sources have associated genetic variants of *UGT1A* genes with ulcerative colitis and IBD in humans [21,22,23,24], functionally implicating decreased expression of these proteins during inflammatory states in disease. Erdmann and colleagues suggest inflammatory processes occurring during IBD may alter the expression of UGT proteins, as their expression is negatively correlated with several inflammatory cytokines [22]. The Reactome functional network, generated from the IBD dataset proteins (Figure 11), suggests that the UGT proteins hypothetically interact as a distinct group, however this is driven only by the homology between the proteins. The expression of these genes is tissue-specific, as identified within the GTeX database, which was not depicted by the functional network. While the UGT genes are clearly an independent module in the Reactome network, they are linked to the rest of the network by the CEBPB transcription factor interaction with *UGT1A10*. The IBD GRNs propose a new directional interaction between the UGT- and non-UGT genes in IBD patients, a novel finding which necessitates experimental validation. In contrast to the IBD GRN, the PDAC network shares a high number of connections with a predicted, condition- and tissue-agnostic protein–protein interaction (PPI) network involving these proteins (Figure 10). The PPI network is tightly connected (*p* < 5 × 10^−15^), including the core GRN proteins, *SULF1* and *THBS2*, having 3 and 7 connections respectively. The THBS2-INHBA interaction is validated (Figure 6a–c) as is the *SULF1*—*FN1* interaction (Figure 6a–c). The link between *SULF1* and *CAPG* is not revealed in the Reactome functional interaction network or the STRING network created from PDAC genes (Figure 10 and Figure 12), suggesting the need for experimental validation.

The PDAC PPI network (Figure 10) suggests an interaction between *SULF1* and *FN1* that is modeled in the GRN, however this is absent in the Reactome functional network, Figure 12. This disagreement between interaction networks from different databases could be explained by the tissue-specific nature of gene regulation. The GRN proposed in this work captures the *SULF1*-*FN1* interaction missing from the Reactome network and proposes several other novel interactions which may be mediated by the specific disease state (PDAC) and tissue (pancreatic) unique to the network. The PDAC network’s functions (Figure S8) include several cancer specific roles, including regulating senescence and autophagic processes, tumorigenic pathways, and increased *TGF*-β cell-cell signaling, a hallmark of cancer cell differentiation in pancreatic cancer [25]. *SULF1* and *THBS2* have both been recently suggested as core regulators of gene co-expression networks in pancreatic cancer [26]. *THBS2* has been identified as a diagnostic biomarker and downstream target for various pancreatic cancers in humans and mice, highlighting its central role in the proposed PDAC specific GRN [27,28,29]. The *SULF2* protein is expressed in pancreatic cancer cells; both *SULF1* and its homolog *SULF2* have been shown to deferentially splice to regulate pancreatic tumor progression and have been proposed as both biomarkers and treatment targets [30,31,32,33]. While this evidence does not directly validate the downstream interactions of *SULF1* and *THBS2*, proposed by the PDAC GRN, it does reinforce the centrality of these core regulators to the PDAC specific biological processes which the GRNs regulate.

## 4. Materials and Methods

In order to infer a gene regulatory network (GRN) and identify the potential regulatory genes and target genes, we have used two different general-purpose, open-source, algorithms designed to be applied to any type of network data, namely the PLSNET (Partial Least Squares NETwork) [14] algorithm and a modified version of the MIDER (Mutual Information Distance and Entropy Reduction) [2] algorithm. The two algorithms employ different techniques to generate a GRN. PLSNET is an ensemble method that expresses the GRN inference problem as a feature selection problem. MIDER, on the other hand, uses the statistic features of the data. In order to quantify how well the generated GRN corresponds to the gene expression profile, we used the computational framework developed by Kotiang and Eslami [19].

### 4.1. Datasets and Gene Selection

We have used three different gene expression datasets, including one RNA sequencing dataset resulting from inflammatory bowel disease (IBD) [15], and two microarray datasets resulting from pancreatic ductal adenocarcinoma (PDAC), and acute myeloid leukaemia (AML) studies respectively (See Table 4 for information). The IBD dataset consists of 20 patients, 10 with UC (ulcerative colitis) and 10 with PSC-IBD (primary sclerosing cholangitis—inflammatory bowel disease). Quraishi et al. [15] provide the RNA library preparation and differential gene expression analysis methods followed. The PDAC dataset includes 36 each cases and controls, *n* = 36 cases), accession GSE15471 [16]. The AML dataset is from a three-cohort study of acute myeloid leukaemia (AML) cell lines with *n* = 404 AML samples and *n* = 138 control samples GSE15061 [17]. To compute a case/control study, a third transitional cohort of MDS samples were excluded from analyses. Each microarray dataset obtained from author-submitted Robust Multichip Average normalized Affymetrix chips, and processed as described in [34]. Briefly, to obtain significantly differential expressed genes elastic net and lasso models were repeatedly run to classify cell libraries by condition, and after 100 repetitions using varying case/control splits features were ranked by how often they influenced model performance as denoted by the β coefficient in lasso/ridge regression. Genes appearing in >80 models were considered stable influential biomarkers and retained for input into downstream analysis. More specifically, for each dataset, the input data was first split into training and testing sets (75:25), with the binary outcome (case/control). Then two types of penalized logistic regression models (LASSO and Elastic Net (EN)) were applied in combination with glmnet function. Penalized methods impose a penalty on regression coefficients, with LASSO [35] and EN [36] allowing for a continuous shrinkage of the coefficients towards and including 0, and therefore allow for an automatic variable selection as well. The glmnet function, according to Equation (1), necessitates two parameters, namely alpha and lambda.

(1) $$\overset{n}{\underset{i=1}{\sum }}{\left(y_{i}-\beta_{0}-\overset{p}{\underset{j=1}{\sum }}\beta_{j}x_{ij}\right)}^{2}+\lambda \left[\frac{\left(1-\alpha \right){∥\beta ∥}_{2}^{2}}{2}+\alpha {∥\beta ∥}_{1}\right]$$

We set alpha = 1 for LASSO and 0.5 for Elastic Net, to reflect LASSO being a more stringent method, with coefficients shrinking quicker towards 0, and EN allowing for the selection of grouped or correlated variables [36]. For each modeling approach, a 10-fold cross validation was performed to optimize for the regularization parameter lambda. Both models were then fitted to the data and the process was repeated 100 times, randomly splitting between the training and the testing sets for each model. We then selected only those features with 80% or above frequency of appearance.

Finally, we have also performed our analysis on the benchmark DREAM4 in silico network challenge (http://wiki.c2b2.columbia.edu/dream/index.php/D4c2). This network inference challenge is aimed at reverse engineering gene networks of sizes 10 and 100, respectively. These networks are artificially generated as reported in [37] and have no biological interpretations.

### 4.2. PLSNET

PLSNET [14] is an ensemble gene regulatory network inference method that decomposes the inference problem with p genes into p subproblems. Each subproblem is then solved independently using partial least squares (PLS) based on a feature selection algorithm. Let $D\;=\;\left[x_{1},x_{1},\ldots ,x_{p}\right]\in {\mathbb{R}}^{n\times p}$ be the gene expression data, where $x_{1}$ is a column vector of expression values of i−th gene in n experimental conditions. Then the feature selection problem is defined in Equation (2) as:

(2) $$x_{i}=f\left(x^{-i}\right)+\epsilon ,\forall i\;\in \;\left\{1,2,\ldots ,p\right\},$$

where $x^{-i}\;=\;\left[x_{1},x_{1},\ldots ,x_{i-1},x_{i+1},\ldots ,x_{p}\right]$ are the potential regulator genes and f is a regression function that exploits the expression $x^{-i}$. Usually, f can be defined using Equation (3) as:

(3) $$f\left(x^{-i}\right)\;=\;\underset{j}{\sum }w_{ji}x_{j},\forall i\;\in \;\left\{1,2,\ldots ,p\right\},$$

where $w_{ji}\geq 0$ represents the strength that gene i regulates gene j.

The final result is improved under the assumption that if a regulatory gene regulates many target genes (e.g., the regulatory gene is hub node), it is an important regulatory gene. Finally, the regulatory genes are scored based on their impacts on multiple target genes. The output of the PLSNET is a weighted adjacency matrix w, where $w_{ij}$ represents the strength that gene i regulates gene j. The input to PLSNET requires three additional parameters, namely m, k, and T. Here, m represents the number of components, k represents the number of regulatory genes, and T is the number of iterations. Finally, a threshold value is used to select important interactions between the nodes. Interactions with a weight less than a certain threshold value are discarded.

### 4.3. MIDER

MIDER [2] constructs a GRN based on statistical features of the data. It uses mutual information and conditional entropy computed from the gene expression profile to estimate the likelihood of an interaction between two genes. The MIDER framework adopts the following steps to infer a GRN from gene expression profile.

1. The algorithm estimates a number of statistical properties, including conditional entropies, transfer entropies, and mutual information from the data. These estimates are then employed at different stages of the network construction. Let X be a discrete random variable with alphabet χ and probability mass function $p\left(x\right)$. Then the entropy is defined using Equation (4) as:
  (4) $$H\left(X\right)=-\underset{x\in \chi }{\sum }p\left(x\right)\log\left(p\left(x\right)\right)$$
  For a continuous variable ∑ is replaced by ∫. The joint entropy of two random variables X and Y is defined using Equation (5) as:
  (5) $$H\left(X,Y\right)=-\underset{x}{\sum }\underset{y}{\sum }p\left(x,y\right)\log\left(p\left(x,y\right)\right)$$
  The conditional probability of a random variable X conditioning upon another random variable Y is defined using Equation (6) as:
  (6) $$H\left(Y|X\right)=-\underset{x}{\sum }\underset{y}{\sum }p\left(x,y\right)\log\left(p\left(y|x\right)\right).$$
  Finally, the mutual information between two random variables is defined using Equation (7) as:
  (7) $$I\left(X,Y\right)=H\left(X\right)-H\left(Y|X\right)=H\left(X\right)+H\left(Y\right)-H(X,Y)$$
2. Based on the mutual information estimates, a distance matrix between all the genes variables is constructed. The distance between two variables X and Y is computed as $d\left(X,Y\right)=e^{-I\left(X,Y\right)}$. This distance matrix is used as a first approximation of the connections between variables. Since $I\left(X,Y\right)$ is symmetric, the distance matrix is also symmetric, i.e., $d\left(X,Y\right)=d\left(Y,X\right)$.
3. An entropy reduction, based on conditional entropies, is then applied to further refine the map. This allows for the discriminating between direct and indirect connections. One of the limitations of entropy reduction is that it requires a large amount of data to get a reliable estimate [38]. Instead of considering all the reactants, MIDER performs a limited reconstruction by considering only first m important ones. In the MATLAB implementation of MIDER [2] algorithm, the authors have estimated joint entropies of up to 4-tuples of variables. In our case, since we have a limited number of samples, we have used only 3-tuples of variables (i.e., a value of m=2). This is also the default value used in the implementation of MIDER for computation reasons.
4. Finally, the directions of the inferred links are assigned using transfer entropy, $T_{X\to Y}$, which is a non-symmetric measure of causality [39]. Here, for every predicted link, MIDER calculates two transfer entropies (i.e., $T_{X\to Y}$ and $T_{Y\to X}$) and assigns the causality in the direction corresponding to the maximum of the two.

MIDER also requires the initialisation of different parameter values prior to its execution. For our experiments, we have used the default parameter values used in the original implementation of MIDER. Unlike many other gene inference methods (such as ARACNE [7], MRNET [8], and CLR [3]), MIDER also infers the directionality of the interaction. It uses the concept of transfer entropy to identify the direction of the link. However, in some cases, the algorithm may not be able to accurately predict the directionality for a number of reasons, including the algorithm’s inability to compute the transfer entropies when the number of samples are small. Furthermore, Villaverde et al. [2] have only evaluated their framework on undirected networks (or ignored the direction of the networks). In our work, instead of using transfer entropies to determine the direction of an interaction, we make use of the mutual information and conditional entropies computed in the 2nd and 3rd steps of the algorithm.

### 4.4. System Wide Analysis of GRN

To analyse and computationally validate the GRNs, produced by applying PLSNET and MIDER to the gene expression profile data, we apply the computational framework developed by Kotiang and Eslami [19]. This framework takes as an input a GRN and uses the popular Loopy Belief Propagation (or sum-product message passing) algorithm [18] to predict the gene’s states. The following steps are performed.

1. The first step is to convert the Bayesian gene network into an equivalent factor graph. A factor graphs is a bipartite graph with two types of nodes, i.e., a variable node that denotes each random variable and a factor node that denotes a local function.
2. The next step is to discretise the data. For a GRN, this is considered an integral part of the model and is usually performed for computational efficiency. Without discretisation, a large amount of data is required to accurately learn the regulatory relations [40]. Furthermore, discretisation helps reduce noise in the continuous variables [40]. In the framework developed by Kotiang and Eslami [19], this step is done by using a Gaussian mixture model with different (at least two) quantisation levels.
3. To approximate the marginal posterior distributions across all genes, the loopy-belief propagation (LBP) algorithm is applied. LBP is a popular message passing algorithm that can be used to infer probabilities in a loopy graph. It is an iterative procedure that minimises the Bethe free energy [18] and achieves a good approximation if the solution converges in fixed number of iterations [41].
4. Finally, the predicted marginals are compared with node proportions to estimate the performance of the inferred GRN.

Identifying an optimal discretisation is a NP-Complete problem [42]. Kotiang and Eslami [19] have suggested to use two or three levels of quantisations. In this work, since the sample size is very small compared to the number of genes, we have used only two levels of quantisation. Furthermore, in all our cases, the LBP converges in very few iterations (less than 100) which suggests that the inferred GRN achieves good approximation to the gene expression profile.

### 4.5. Ensemble Approach

In order to infer the structure of a GRN with higher accuracy, here we propose an ensemble-based model that combines the output of PLSNET and MIDER. We have empirically demonstrated that our proposed ensemble-based approach not only successfully outputs a GRN that can be computationally validated, but it also identifies the potential key regulators within the data. The approach first applies PLSNET with different threshold values and identifies the key regulators in the network. Next, MIDER is applied to infer the GRN structure. This GRN is further refined by removing all the false-positive edges identified by PLSNET. The final network is validated using the framework developed by Kotiang and Eslami [19] that uses the LBP [18] algorithm to predict the gene’s states. All the codes were implemented in MATLAB (v. R2020b).

### 4.6. Biological Investigation

GRNs from the PDAC and IBD datasets were further interrogated using available biomedical databases as well as background biomedical knowledge. To investigate the biological function of identified GRNs, gene set enrichment analyses were performed using the Gprofiler software suite on 24 September 2020, using default unordered query settings with H. Sapiens selected as species and correcting for multiple testing by the Benjamini-Hochberg method [43,44]. Additionally, genes from each GRN were compared to predicted protein–protein interaction networks from the STRINGdb database, version 10 using default settings [45]. Genes belonging to each GRN were also submitted to functional network analysis with the ReactomeFI-Viz plugin v. 7.2.3 in Cytoscape v. 3.9 [46,47].

## Figures and Tables

**Table 1:** Frequencies of different genes appearing as Regulatory (R), Target (T), or Intermediate (I) gene for different threshold values for the IBD data. For each threshold value, the experiment was executed 100 times with the same set of parameter values.

Genes	Top 2%			Top 5%			Top 10%			Top 15%			Top 20%		
	R	T	I	R	T	I	R	T	I	R	T	I	R	T	I
*TUBB2A*	0	66	0	0	98	0	0	100	0	0	100	0	0	100	0
*CALU*	0	36	0	0	97	0	0	100	0	0	100	0	0	100	0
*USP2*	0	1	0	0	23	0	0	98	0	0	100	0	0	100	0
*UGT1A1*	0	0	0	1	0	0	1	14	0	0	84	2	0	90	0
*ASS1*	0	49	0	0	94	0	0	100	0	0	100	0	0	100	0
*UGT1A6*	7	0	0	26	1	0	86	1	2	87	0	12	23	0	77
*UGT1A10*	81	1	0	99	1	0	99	0	1	92	0	8	33	0	67
*UGT1A9*	82	0	0	95	0	0	97	0	2	95	0	5	59	0	41
*UGT1A7*	5	0	0	12	0	0	40	3	0	46	12	37	0	1	99
*TRIM29*	0	31	0	0	93	0	0	100	0	0	100	0	0	100	0
*PITX2*	0	99	0	0	100	0	0	100	0	0	100	0	0	100	0
*TSTA3*	0	3	0	0	45	0	0	96	0	0	100	0	0	100	0
*PCSK1*	0	20	1	0	75	1	0	99	1	0	99	1	0	99	1
*CXCL1*	0	2	0	1	28	0	0	88	3	0	96	4	0	94	6
*CCL13*	0	27	0	0	95	0	0	100	0	0	100	0	0	100	0
*TNIP3*	1	2	0	2	26	0	1	85	1	0	98	2	0	97	3

**Table 2:** Distribution of the posteriors versus observed experimental states for the IBD dataset.

Genes	Predicted Marginals		Observed States	
	0	1	0	1
*TUBB2A*	0.55	0.45	0.55	0.45
*CALU*	0.9	0.1	0.9	0.1
*USP2*	0.2	0.8	0.2	0.8
*UGT1A1*	0.5498	0.4502	0.55	0.45
*ASS1*	0.9	0.1	0.9	0.1
*UGT1A6*	0.5999	0.4001	0.6	0.4
*UGT1A10*	0.5999	0.4001	0.6	0.4
*UGT1A9*	0.5998	0.4002	0.6	0.4
*UGT1A7*	0.3501	0.6499	0.35	0.65
*TRIM29*	0.45	0.55	0.45	0.55
*PITX2*	0.5	0.5	0.5	0.5
*TSTA3*	0.75	0.25	0.75	0.25
*PCSK1*	0.85	0.15	0.85	0.15
*CXCL1*	0.35	0.65	0.35	0.65
*CCL13*	0.25	0.75	0.25	0.75
*TNIP3*	0.7	0.3	0.7	0.3

**Table 3:** Frequencies of different genes appearing as Regulatory (R), Target (T), or Intermediate (I) gene for different threshold values for the PDAC dataset.

Genes	Top 2%			Top 5%			Top 10%			Top 15%			Top 20%		
	R	T	I	R	T	I	R	T	I	R	T	I	R	T	I
*SULF1*	100	0	0	100	0	0	65	0	35	3	0	97	0	0	100
*COL8A1*	63	1	0	6	5	89	0	1	99	0	0	100	0	0	100
*INHBA*	0	0	0	2	5	0	3	69	24	0	29	71	0	4	96
*FN1*	7	0	0	29	27	30	0	4	96	0	0	100	0	0	100
*COL10A1*	0	0	0	0	98	0	0	97	3	0	69	31	0	18	82
*THBS2*	100	0	0	100	0	0	100	0	0	76	0	24	14	0	86
*NTM*	0	0	0	0	8	0	0	100	0	0	94	6	0	80	20
*NOX4*	0	0	0	21	31	15	0	13	87	0	1	99	0	0	100
*RASAL2*	0	0	0	0	0	0	0	75	0	0	100	0	0	100	0
*ADAMTS12*	0	0	0	0	12	0	0	98	2	0	49	51	0	17	83
*CAPG*	0	96	0	0	100	0	0	100	0	0	100	0	0	95	5
*LTBP1*	0	9	0	0	98	0	0	100	0	0	100	0	0	100	0
*CTHRC1*	0	0	0	0	2	0	0	78	0	0	100	0	0	100	0
*FAP*	0	13	0	0	78	21	0	24	76	0	2	98	0	0	100
*WISP1*	26	0	0	61	8	27	0	0	100	0	0	100	0	0	100
*VCAN*	2	0	0	15	0	0	36	11	41	0	4	96	0	0	100
*TIMP1*	0	0	0	0	66	0	0	100	0	0	88	12	0	39	61
*MIR34AHG*	0	100	0	0	100	0	0	100	0	0	100	0	0	100	0
*SLPI*	0	100	0	0	100	0	0	100	0	0	100	0	0	100	0
*GPRC5A*	0	100	0	0	100	0	0	100	0	0	100	0	0	100	0

**Table 4:** Information about all the real-world datasets used in this study. Here, N represents the number of genes used for network inference.

Author Name	Disease Type	N	Reference
Quraishi et al.	Inflammatory bowel disease	16	[15]
Rajamani et al.	Pancreatic ductal adenocarcinoma	20	[16]
Mills et al.	Acute myeloid leukemia	60	[17]

## Supplementary Materials

Code can be found at https://github.com/azizfurqan/PGM. The following are available online at https://www.mdpi.com/1422-0067/21/21/7886/s1. Figure S1: GRN generated from the application of PLSNET on the IBD dataset. Figure S2: The two identified regulators are represented by the largest observed effect size. Figure S3: GRN generated using PLSNET for AML. The threshold value is selected in such a way that the top 10% of all edges are selected. Figure S4: Pearson correlation plots for the AML dataset. Figure S5: GRN generated from the application of MIDER on the DREAM dataset. Figure S6: Pearson correlation plots for the DREAM4. Figure S7: Gene set enrichment results from the IBD dataset. Figure S8: Gene set enrichment results from the PDAC dataset. Table S1: Distribution of the posteriors versus observed experimental states for GSE15471. Table S2: Distribution of the posteriors versus observed experimental states in the AML dataset. Table S3: Distribution of the posteriors versus observed experimental states in the AML dataset. Table S4: Frequencies of different genes appearing as Regulatory (R), Target (T), or Intermediate (I) genes for different threshold values in the DREAM4 dataset. Table S5: Distribution of the posteriors versus observed experimental states for DREAM4.

## Abbreviations

AML	Acute Myeloid Leukemia
GRN	Gene Regulatory Network
IBD	Inflammatory Bowel Disease
LBP	Loopy Belief Propagation
MIDER	Mutual Information Distance and Entropy Reduction
PDAC	Pancreatic Ductal Adenocarcinoma
PLSNET	Partial Least Square based Network
