# Supplementary material for: Biomarker Prioritisation and Power Estimation Using Ensemble Gene Regulatory Network Inference
Source: Int J Mol Sci. 2020 Oct 23;21(21):7886. doi: 10.3390/ijms21217886 (PMC7660606; doi:10.3390/ijms21217886)
Supplement: Supplementary file 1 [file ijms-21-07886-s001.pdf]

International Journal of
Molecular Sciences

Article

Biomarker Prioritisation and Power Estimation Using Ensemble Gene Regulatory Network Inference

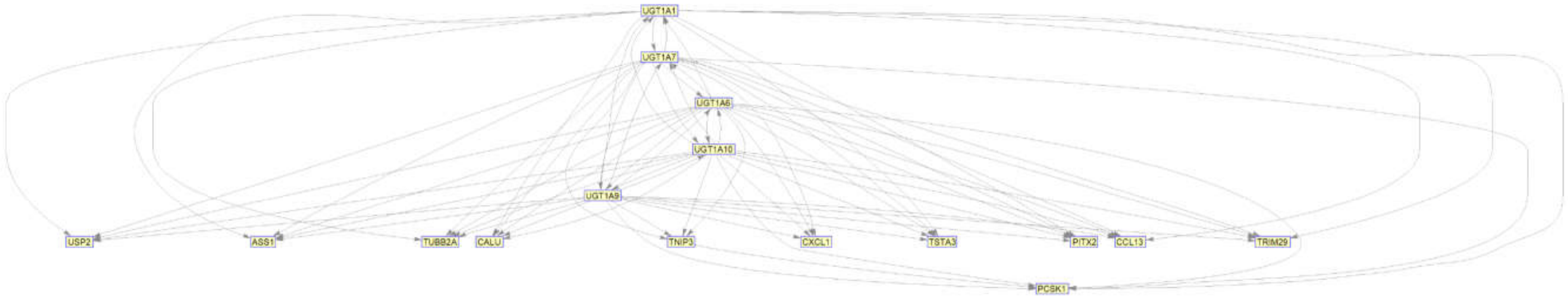

Figure S1. GRN generated from the application of PLSNET on the IBD dataset. The threshold value is selected in such a way that the top 30% edges are selected. UGT genes are delineating as regulators for other genes.

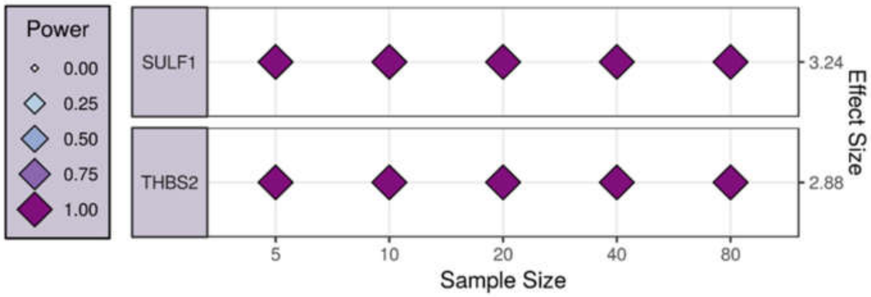

Figure S2. The two identified regulators are represented by the largest observed effect size. The effect size of each assessed variable is shown along the y axis and a series of sample sizes along the x axis.

Table S1. Distribution of the posteriors versus observed experimental states for GSE15471.

Genes	Predicted Marginals		Observed States	
	**0**	**1**	**0**	**1**
SULF1	0.6026	0.3974	0.6026	0.3974
COL8A1	0.4103	0.5897	0.4103	0.5897
INHBA	0.6026	0.3974	0.6026	0.3974
FN1	0.6026	0.3974	0.6026	0.3974
COL10A1	0.5637	0.4363	0.5641	0.4359
THBS2	0.5641	0.4359	0.5641	0.4359
NTM	0.6021	0.3979	0.6026	0.3974
NOX4	0.3846	0.6154	0.3846	0.6154
RASAL2	0.5385	0.4615	0.5385	0.4615
ADAMTS12	0.5769	0.4231	0.5769	0.4231
CAPG	0.6667	0.3333	0.6667	0.3333
LTBP1	0.6538	0.3462	0.6538	0.3462
CTHRC1	0.5128	0.4872	0.5128	0.4872
FAP	0.5769	0.4231	0.5769	0.4231
WISP1	0.6538	0.3462	0.6538	0.3462
VCAN	0.4235	0.5765	0.4231	0.5769
TIMP1	0.5641	0.4359	0.5641	0.4359
MIR34AHG	0.8462	0.1538	0.8462	0.1538
SLPI	0.5513	0.4487	0.5513	0.4487
GPRC5A	0.6406	0.3594	0.641	0.359

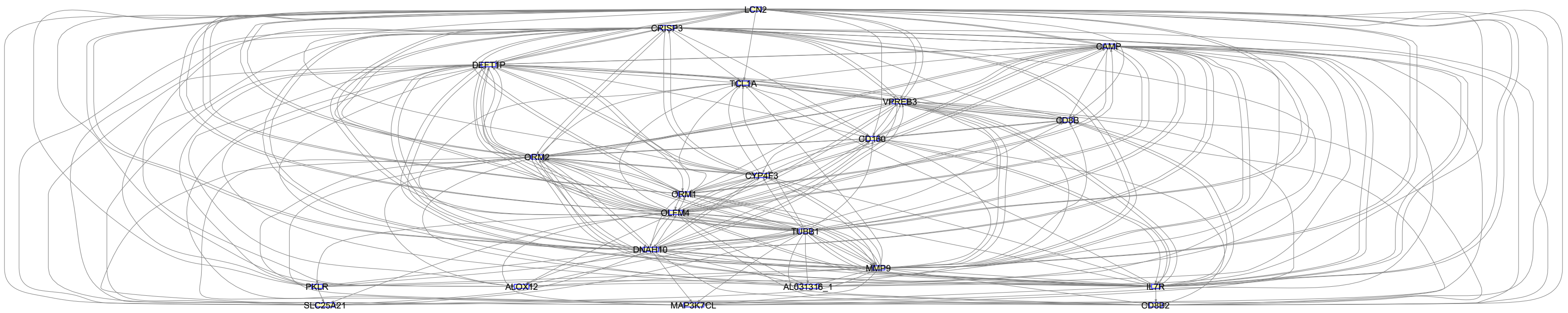

Figure S3. GRN generated using PLSNET for AML. The threshold value is selected in such a way that the top 10% of all edges are selected. This figure does not show Table 22. genes that do not have any interactions. These genes include *HHEX*, *KLF10*, *SOX4*, *MAPK12*, *TRIO*, *GAS5*, *SOCS5*, *MARK4*, *ZNF469*, *EEF1A1*, *C6orf48*, *ASPH*, *SLC17A9*, *RPL22*, *RAB37*, *FBXL19*, *RTN2*, *MSRB3*, *C5*, *SNX33*, *FLT3*, *MERTK*, *CD44*, *ITPR1*, *CRTC1*, *IGF2BP2*, *URB1_AS1*, and 'ARRDC4'.

Table S2. Distribution of the posteriors versus observed experimental states in the AML dataset.

Genes	Top 2%			Top 5%			Top 10%			Top 15%			Top 20%		
	**R**	**T**	**I**	**R**	**T**	**I**	**R**	**T**	**I**	**R**	**T**	**I**	**R**	**T**	**I**
LCN2	0	24	76	0	4	96	0	0	100	0	0	100	0	0	100
HHEX	0	0	0	0	0	0	0	0	0	0	0	0	0	0	0
CRISP3	0	40	60	0	12	88	0	5	95	0	5	95	0	5	95
CAMP	0	6	94	0	1	99	0	0	100	0	0	100	0	0	100
KLF10	0	0	0	0	0	0	0	0	0	0	0	0	0	0	0
SOX4	0	0	0	0	0	0	0	0	0	0	0	0	0	0	0
HOXA9	0	0	0	0	0	0	0	2	0	0	2	0	0	2	0
MAP3K7CL	0	93	0	0	97	3	0	88	12	0	88	12	0	88	12
MAPK12	0	0	0	0	0	0	0	0	0	0	0	0	0	0	0
TRIO	0	0	0	0	0	0	0	0	0	0	0	0	0	0	0
DEFT1P	0	76	24	0	37	63	0	15	85	0	15	85	0	15	85
GAS5	0	0	0	0	0	0	0	0	0	0	0	0	0	0	0
BMP1	0	0	0	0	1	0	0	1	0	0	1	0	0	1	0
CD8B2	0	83	10	0	54	46	0	28	72	0	28	72	0	28	72
SOCS5	0	0	0	0	0	0	0	0	0	0	0	0	0	0	0
PTH2R	0	2	0	0	28	0	0	61	0	0	61	0	0	61	0
MARK4	0	0	0	0	0	0	0	0	0	0	0	0	0	0	0
FSCN1	0	0	0	0	0	0	0	1	0	0	1	0	0	1	0
CYP4F3	0	46	54	0	11	89	0	2	98	0	2	98	0	2	98
ZNF469	0	0	0	0	0	0	0	0	0	0	0	0	0	0	0
DNAH10	0	63	37	0	17	83	0	7	93	0	7	93	0	7	93
HOXA10	0	0	0	0	1	0	0	3	0	0	3	0	0	3	0
VPREB3	0	53	47	0	13	87	0	3	97	0	3	97	0	3	97
EEF1A1	0	0	0	0	0	0	0	0	0	0	0	0	0	0	0
C6orf48	0	0	0	0	0	0	0	0	0	0	0	0	0	0	0
ASPH	0	0	0	0	0	0	0	0	0	0	0	0	0	0	0
MMP9	0	45	55	0	8	92	0	3	97	0	3	97	0	3	97
SLC17A9	0	0	0	0	0	0	0	0	0	0	0	0	0	0	0
UBE2E2	0	0	0	0	0	0	0	1	0	0	1	0	0	1	0
RPL22	0	0	0	0	0	0	0	0	0	0	0	0	0	0	0
NETO2	0	0	0	0	0	0	0	1	0	0	1	0	0	1	0
OLFM4	0	61	39	0	18	82	0	5	95	0	5	95	0	5	95
IL7R	0	71	28	0	32	68	0	7	93	0	7	93	0	7	93
PNKD	0	0	0	0	0	0	0	1	0	0	1	0	0	1	0
ORM1	0	30	70	0	6	94	0	0	100	0	0	100	0	0	100
RAB37	0	0	0	0	0	0	0	0	0	0	0	0	0	0	0
TUBB1	2	68	4	0	62	38	0	28	72	0	28	72	0	28	72
FBXL19	0	0	0	0	0	0	0	0	0	0	0	0	0	0	0
RTN2	0	0	0	0	0	0	0	0	0	0	0	0	0	0	0

MSRB3	0	0	0	0	0	0	0	0	0	0	0	0	0	0	0
C5	0	0	0	0	0	0	0	0	0	0	0	0	0	0	0
CFD	0	0	0	0	0	0	0	2	0	0	2	0	0	2	0
ORM2	0	43	57	0	14	86	0	6	94	0	6	94	0	6	94
SNX33	0	0	0	0	0	0	0	0	0	0	0	0	0	0	0
FLT3	0	0	0	0	0	0	0	0	0	0	0	0	0	0	0
AL022313_1	0	0	0	0	1	0	0	4	0	0	4	0	0	4	0
MERTK	0	0	0	0	0	0	0	0	0	0	0	0	0	0	0
CD160	4	65	26	0	24	76	0	8	92	0	8	92	0	8	92
ALOX12	0	75	0	0	92	8	0	70	30	0	70	30	0	70	30
PKLR	2	78	8	0	46	54	0	19	81	0	19	81	0	19	81
CD44	0	0	0	0	0	0	0	0	0	0	0	0	0	0	0
CD8B	7	49	31	0	23	77	0	5	95	0	5	95	0	5	95
TCL1A	0	77	23	0	36	64	0	11	89	0	11	89	0	11	89
ITPR1	0	0	0	0	0	0	0	0	0	0	0	0	0	0	0
AL031316_1	0	97	3	0	72	28	0	43	57	0	43	57	0	43	57
CRTC1	0	0	0	0	0	0	0	0	0	0	0	0	0	0	0
IGF2BP2	0	0	0	0	0	0	0	0	0	0	0	0	0	0	0
URB1_AS1	0	0	0	0	0	0	0	0	0	0	0	0	0	0	0
SLC25A21	0	99	0	0	91	9	0	78	22	0	78	22	0	78	22
ARRDC4	0	0	0	0	0	0	0	0	0	0	0	0	0	0	0

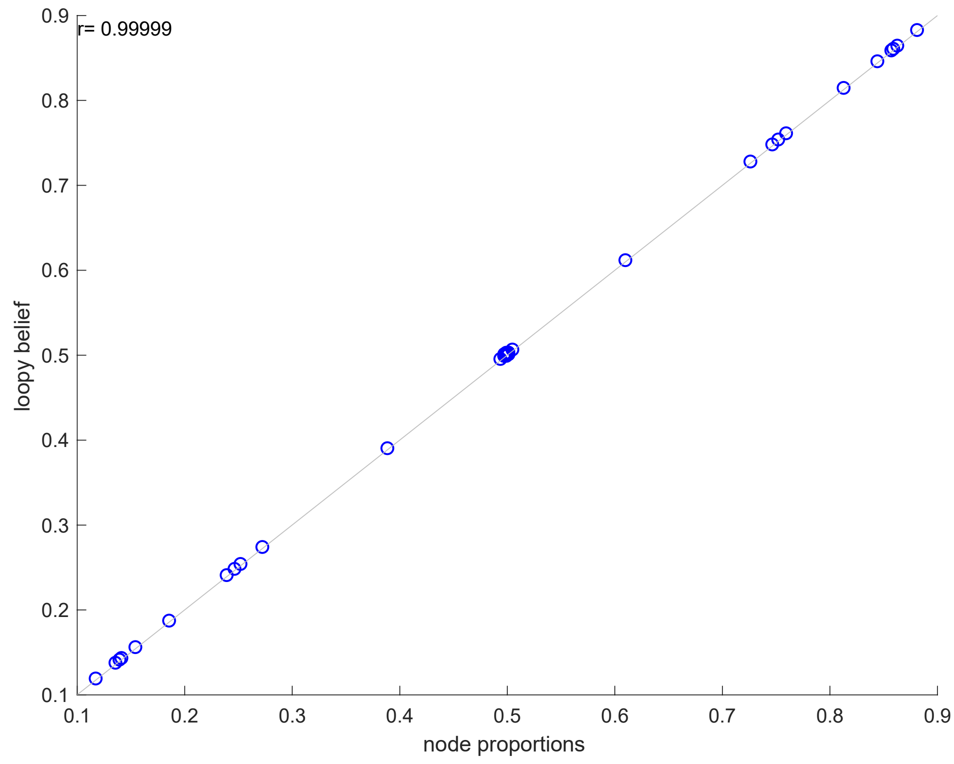

Figure S4. Pearson correlation plots for the AML dataset.

Table S3. Distribution of the posteriors versus observed experimental states in the AML dataset.

**Genes**	**Predicted Marginals**		**Observed States**	
	**0**	**1**	**0**	**1**
LCN2	0.5	0.5	0.5	0.5
HHEX	0.5	0.5	0.4982	0.5018
CRISP3	0.5	0.5	0.5	0.5
CAMP	0.5	0.5	0.5	0.5
KLF10	0.5	0.5	0.5	0.5
SOX4	0.5	0.5	0.5	0.5
HOXA9	0.5	0.5	0.5	0.5
MAP3K7CL	0.1863	0.8137	0.1863	0.8137
MAPK12	0.5	0.5	0.5	0.5
TRIO	0.5	0.5	0.5	0.5
DEFT1P	0.2731	0.7269	0.2731	0.7269
GAS5	0.5	0.5	0.5	0.5
BMP1	0.5	0.5	0.5	0.5
CD8B2	0.8596	0.1404	0.8598	0.1402
SOCS5	0.5	0.5	0.4982	0.5018
PTH2R	0.5	0.5	0.5	0.5
MARK4	0.5	0.5	0.5	0.5
FSCN1	0.5	0.5	0.5	0.5
CYP4F3	0.5	0.5	0.5	0.5
ZNF469	0.5	0.5	0.5	0.5
DNAH10	0.2472	0.7528	0.2472	0.7528
HOXA10	0.5	0.5	0.5	0.5
VPREB3	0.1551	0.8449	0.155	0.845
EEF1A1	0.5	0.5	0.5	0.5
C6orf48	0.5018	0.4982	0.5018	0.4982
ASPH	0.5	0.5	0.5	0.5
MMP9	0.5	0.5	0.5	0.5
SLC17A9	0.5	0.5	0.5	0.5
UBE2E2	0.5	0.5	0.5	0.5
RPL22	0.5	0.5	0.5	0.5
NETO2	0.5	0.5	0.5	0.5
OLFM4	0.6107	0.3893	0.6107	0.3893
IL7R	0.7471	0.2529	0.7472	0.2528
PNKD	0.5	0.5	0.5	0.5
ORM1	0.5	0.5	0.5	0.5
RAB37	0.5	0.5	0.5	0.5
TUBB1	0.5	0.5	0.5	0.5
FBXL19	0.5	0.5	0.5	0.5
RTN2	0.5	0.5	0.5	0.5
MSRB3	0.4945	0.5055	0.4945	0.5055

C5	0.4982	0.5018	0.4982	0.5018
CFD	0.5	0.5	0.5	0.5
ORM2	0.7601	0.2399	0.7601	0.2399
SNX33	0.5	0.5	0.5	0.5
FLT3	0.5	0.5	0.5	0.5
AL022313_1	0.5018	0.4982	0.5	0.5
MERTK	0.5018	0.4982	0.5018	0.4982
CD160	0.1367	0.8633	0.1365	0.8635
ALOX12	0.5	0.5	0.5	0.5
PKLR	0.5	0.5	0.5	0.5
CD44	0.5	0.5	0.5	0.5
CD8B	0.1422	0.8578	0.1421	0.8579
TCL1A	0.8818	0.1182	0.8819	0.1181
ITPR1	0.5	0.5	0.5	0.5
AL031316_1	0.5	0.5	0.5	0.5
CRTC1	0.5	0.5	0.5	0.5
IGF2BP2	0.4982	0.5018	0.4982	0.5018
URB1_AS1	0.5018	0.4982	0.5018	0.4982
SLC25A21	0.5	0.5	0.5	0.5
ARRDC4	0.5	0.5	0.5	0.5

Table S4. Frequencies of different genes appearing as Regulatory (R), Target (T), or Intermediate (I) genes for different threshold values in the DREAM4 dataset.

**Genes**	**Top 2%**			**Top 5%**			**Top 10%**			**Top 15%**			**Top 20%**		
	**R**	**T**	**I**	**R**	**T**	**I**	**R**	**T**	**I**	**R**	**T**	**I**	**R**	**T**	**I**
G1	0	0	0	8	21	1	1	0	99	0	0	100	0	0	100
G2	0	0	0	0	0	0	0	69	0	0	100	0	0	100	0
G3	0	0	0	0	0	0	0	88	0	0	99	0	0	100	0
G4	0	0	0	0	0	0	0	29	0	0	99	0	0	100	0
G5	0	0	0	21	8	1	0	1	99	0	0	100	0	0	100
G6	1	3	0	2	8	90	0	0	100	0	0	100	0	0	100
G7	0	0	0	0	79	0	0	100	0	0	100	0	0	92	8
G8	3	1	0	8	2	90	0	0	100	0	0	100	0	0	100
G9	98	0	0	100	0	0	100	0	0	100	0	0	82	0	18
G10	0	96	0	0	100	0	0	100	0	0	100	0	0	100	0

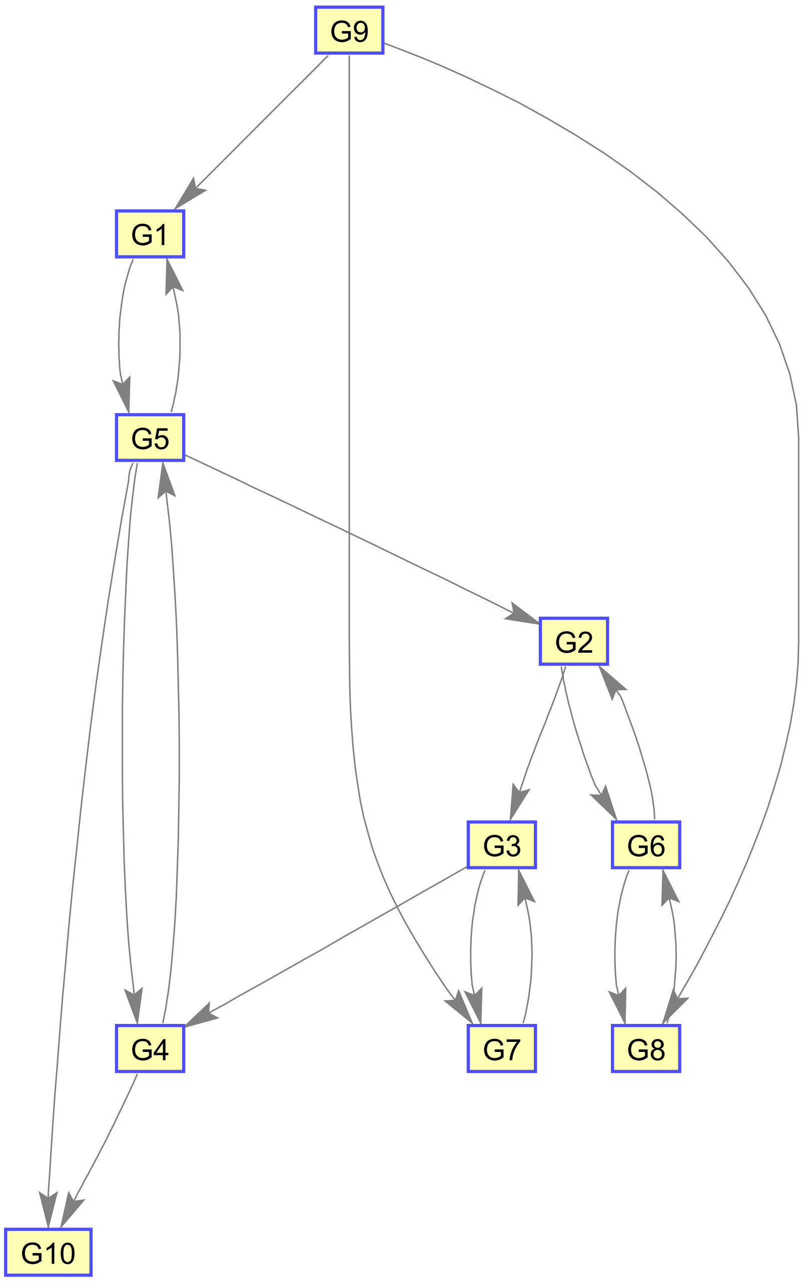

Figure S5. GRN generated from the application of MIDER on the DREAM dataset.

Table S5. Distribution of the posteriors versus observed experimental states for DREAM4.

Genes	Predicted Marginals		Observed States	
	**0**	**1**	**0**	**1**
G1	0.6026	0.3974	0.6026	0.3974
G2	0.4103	0.5897	0.4103	0.5897
G3	0.6026	0.3974	0.6026	0.3974
G4	0.6026	0.3974	0.6026	0.3974
G5	0.5637	0.4363	0.5641	0.4359
G6	0.5641	0.4359	0.5641	0.4359
G7	0.6021	0.3979	0.6026	0.3974
G8	0.3846	0.6154	0.3846	0.6154
G9	0.5385	0.4615	0.5385	0.4615
G10	0.5769	0.4231	0.5769	0.4231

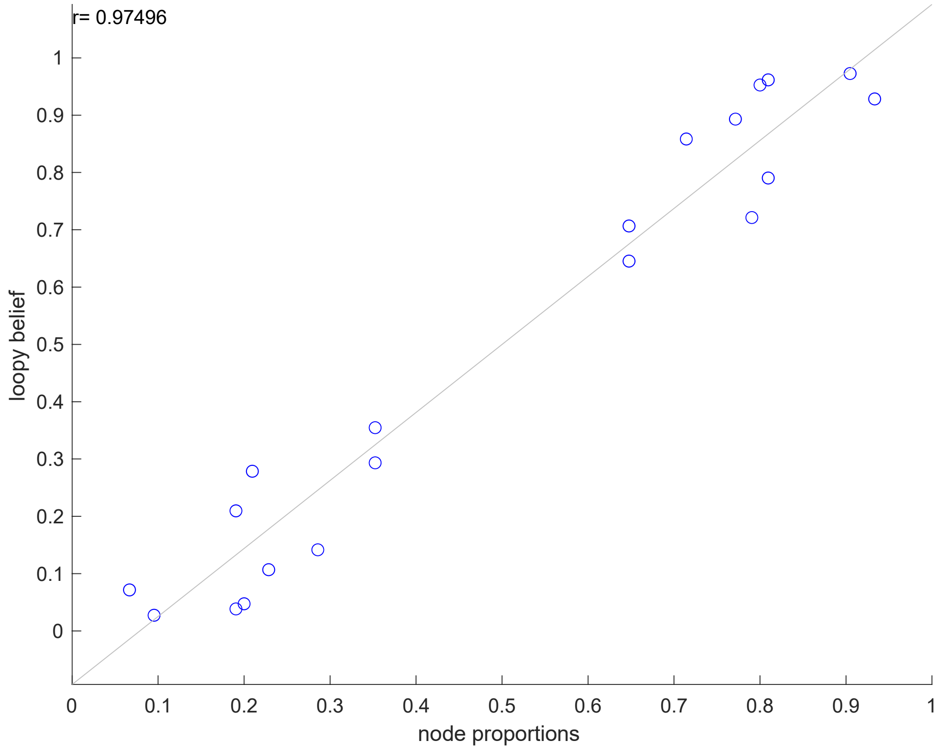

Figure S6. Pearson correlation plots for the DREAM4.

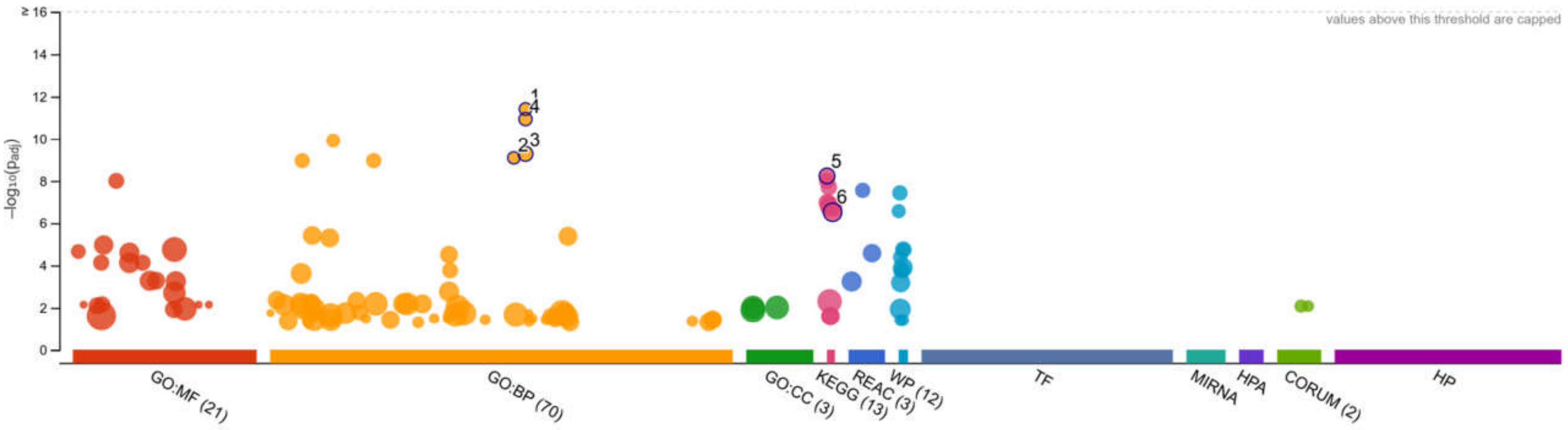

ID	Source	Term ID	Term Name	p_{adj} (query_1)
1	GO:BP	GO:0052696	flavonoid glucuronidation	3.862×10^{-12}
2	GO:BP	GO:0051552	flavone metabolic process	8.236×10^{-10}
3	GO:BP	GO:0052695	cellular glucuronidation	5.314×10^{-10}
4	GO:BP	GO:0052697	xenobiotic glucuronidation	1.158×10^{-11}
5	KEGG	KEGG:00053	Ascorbate and aldarate metabolism	5.858×10^{-9}
6	KEGG	KEGG:04976	Bile secretion	3.061×10^{-7}

**version**	e100_eg47_p14_7733820
**date**	24/09/2020, 12:48:08
**organism**	hsapiens

g:Profiler

Figure S7. Gene set enrichment results from the IBD dataset. Genes were entered into the gProfiler database. P values are FDR adjusted. GO:BP = biological process, KEGG = Kyoto Encyclopedia of Genes and Genomes database.

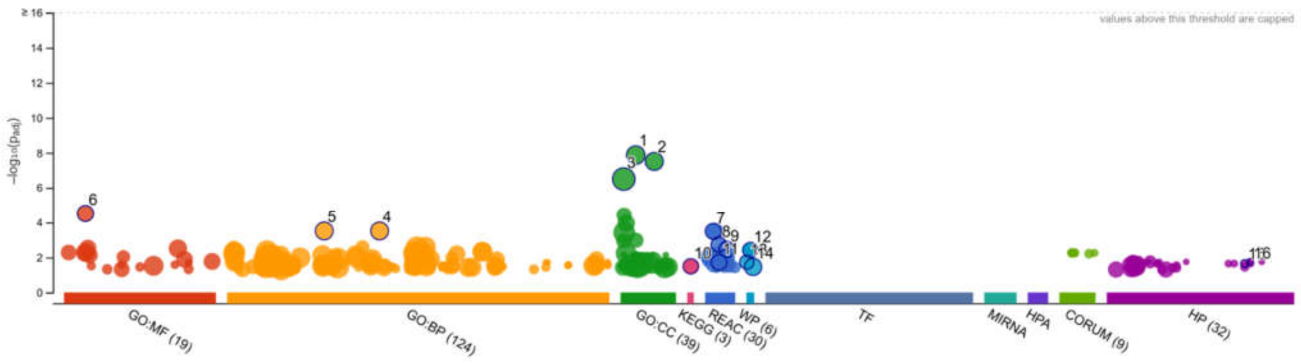

ID	Source	Term ID	Term Name	p_{adj} (query_1)
1	GO:CC	GO:0031012	extracellular matrix	1.343×10^{-8}
2	GO:CC	GO:0062023	collagen-containing extracellular matrix	3.103×10^{-8}
3	GO:CC	GO:0005576	extracellular region	3.121×10^{-7}
4	GO:BP	GO:0043062	extracellular structure organization	3.064×10^{-4}
5	GO:BP	GO:0030198	extracellular matrix organization	3.064×10^{-4}
6	GO:MF	GO:0005201	extracellular matrix structural constituent	3.026×10^{-5}
7	REAC	REAC:R-HSA-14…	Degradation of the extracellular matrix	3.237×10^{-4}
8	REAC	REAC:R-HSA-21…	Integrin cell surface interactions	1.822×10^{-3}
9	REAC	REAC:R-HSA-38…	Regulation of Insulin-like Growth Factor (IGF) tra…	3.478×10^{-3}
10	KEGG	KEGG:04350	TGF-beta signaling pathway	3.220×10^{-2}
11	REAC	REAC:R-HSA-67…	Interleukin-4 and Interleukin-13 signaling	1.897×10^{-2}
12	WP	WP:WP615	Senescence and Autophagy in Cancer	3.811×10^{-3}
13	WP	WP:WP560	TGF-beta Receptor Signaling	1.843×10^{-2}
14	WP	WP:WP4754	IL-18 signaling pathway	3.473×10^{-2}
15	HP	HP:0031030	Elevated carcinoma antigen 125 level	2.260×10^{-2}
16	HP	HP:0031493	Glandular cell neoplasm	1.790×10^{-2}

**version**	e100_eg47_p14_7733820
**date**	23/09/2020, 16:24:50
**organism**	hsapiens

g:Profiler

Figure S8. Gene set enrichment results from the PDAC dataset. Genes were entered into the gProfiler database. *p* values are FDR adjusted. GO:BP = biological process, GO:CC = cellular component, REAC = Reactome database, WP = WikiPathways, HP = Human Phenotype Ontology, KEGG = Kyoto Encyclopedia of Genes and Genomes database.
